# The Influence of Socioeconomic Status on Snacking and Weight among Adolescents: A Scoping Review

**DOI:** 10.3390/nu12010167

**Published:** 2020-01-07

**Authors:** Victoria Guazzelli Williamson, Abhaya Dilip, Julia Rose Dillard, Jane Morgan-Daniel, Alexandra M. Lee, Michelle I. Cardel

**Affiliations:** 1 Department of Psychology, College of Arts and Sciences, University of Oregon, Eugene, OR 97403, USA; 2 Department of Food Science and Human Nutrition, College of Agricultural and Life Sciences, University of Florida, Gainesville, FL 32611, USA; abhaya.dilip@ufl.edu (A.D.); julia.dillard@rockets.utoledo.edu (J.R.D.); 3 Health Science Center Libraries, University of Florida, Gainesville, FL 32610, USA; morgandanie.jane@ufl.edu; 4 Department of Health Outcomes and Biomedical Informatics, College of Medicine, University of Florida, Gainesville, FL 32610, USA; alexlee22@ufl.edu

**Keywords:** review, snacking, obesity, adolescents, socioeconomic status, weight-related outcomes

## Abstract

Eating behaviors, including unhealthy snacking or excessive snacking leading to excess calorie consumption, may contribute to obesity among adolescents. Socioeconomic status (SES) also significantly influences eating behaviors, and low SES is associated with increased risk for obesity. However, little is known regarding the relationship between snacking behavior and SES among adolescents and how this may contribute to obesity-related outcomes. The primary objective of this scoping review was to review the literature to assess and characterize the relationship between SES and snacking in adolescents. The secondary objective was to assess weight-related outcomes and their relation to snacking habits. Included articles were published between January 2000 and May 2019; written in English, Portuguese, or Spanish; and focused on adolescents (13–17 years). In total, 14 bibliographic databases were searched, and seven studies met the inclusion criteria. Preliminary evidence from the seven included studies suggests a weak but potential link between SES and snacking. Additionally, these dietary patterns seemed to differ by sex and income type of country. Finally, only three of the included studies addressed weight-related outcomes, but the overall available evidence suggests that snacking does not significantly affect weight-related outcomes. Due to the small number of included studies, results should be interpreted with caution.

## 1. Introduction

The prevalence of overweight (OW) and obesity (OB) varies throughout the world, with high-income nations having a higher prevalence of OW/OB relative to low-income nations, and differential impacts experienced by persons of various racial, ethnic, and cultural backgrounds [1]. This presents a global public health issue that needs to be addressed, as 50% of school-aged children with obesity continue to have obesity into adulthood [2]. Excess adiposity can result in an increased risk for cardiometabolic diseases [3], some types of cancer [4], and psychological effects such as major depressive disorder and anxiety disorders [3,5].

Obesity is a complex and multifactorial disease that can result from genetic [6], environmental [7], and behavioral contributors [8]. One such contributor is the consumption of excess calories and/or reduced energy expenditure, resulting in positive energy balance. During the transition between childhood and adolescence, a shift in diet composition is observed to include higher rates of snack and soft drink consumption and lower intakes of fruits and vegetables [9]. Alongside increases in the prevalence of obesity [1,10], data from 1977 to 2014 show that snacking in adolescents has increased over the last four decades. It has also been shown that calories from salty snacks, desserts, sweets, and sugar-sweetened beverages continue to be a leading source of calories [11]. While fruit and vegetable intake has decreased, calories provided by these less healthy snacks has increased [12]. Thus, adverse eating behaviors resulting in excess calorie consumption, such as less healthy or frequent snacking, can potentially contribute to the rise of obesity during adolescence.

SES is a strong determinant of an individual’s weight, risk for obesity, and eating behaviors [13]. Individuals with lower SES are more likely to live in disordered and vulnerable neighborhood settings, which are associated with unhealthy food access and consumption [14]. Although rates of obesity have increased across all socioeconomic levels, adolescents who are from families with lower incomes are more likely than those from families with higher incomes to develop obesity [15]. SES, as defined by household income and/or parental education level for adolescents, has the potential to influence food choices [16]. Specifically, SES has been shown to have a strong influence on the consumption of certain food groups over others on a global scale [17,18,19,20], with those with lower SES positions tending to purchase foods that are lower in nutrient density, but high in energy [21]. The relationship between diet and SES may vary by region, however, as diet tends to vary based on country and the income status of that country [22], with the amount and types of food consumed influenced by country of residence [23]. Regardless, SES appears to influence eating behavior, with experimental studies demonstrating that randomizing individuals to lower social status positions results in the consumption of excess calories among adolescents and young adults in the United States, United Kingdom, and Singapore [24,25,26]. This suggests that SES can directly impact eating behavior, thus increasing the risk for obesity and other obesity-related complications. Yet, it remains unclear how SES can impact snacking behaviors and weight status among adolescents, and a review exploring the data characterizing these relationships is needed.

This review assesses the extent to which SES can impact snacking behaviors and weight status among adolescents. The objective of this scoping review is to specifically (1) assess and characterize the relationship between SES and snacking in adolescents (primary) and (2) to assess weight-related outcomes and explore how they differ by SES (secondary). We also explored whether relationships differed by sex or country of origin. Given that there was not a prior review paper written on the effect of SES on snacking and weight-related outcomes among adolescents, we selected to do a scoping review as a preliminary assessment of the potential scope and size of currently available literature. 

## 2. Materials and Methods

In accordance with the Joanna Briggs Institute (JBI) Reviewer’s Manual [27], we developed a protocol and registered the project with the JBI. Registration occurred on 4 July 2019, under the title “The influence of socioeconomic status on snacking and weight among adolescents: A Scoping Review” (listed on http://joannabriggs.org/research/registered_titles.aspx).

A preliminary search for previous systematic reviews, scoping reviews, and protocols on the topic of adolescents’ snacking behavior was conducted on 5 May 2019 using the following sources: Cochrane Database of Systematic Reviews, JBI Database of Systematic Reviews and Implementation Reports, and PROSPERO: International Prospective Register of Systematic Reviews. Of the 77 results retrieved through PROSPERO, none focused on the assessment and characterization of the relationship between SES, snacking, and weight-related outcomes in adolescents.

### 2.1. Identifying the Research Questions 

This scoping review seeks to answer the following questions: (1) How does SES relate to snacking amongst adolescents, and (2) Is snacking associated with weight-related outcomes? 

### 2.2. Inclusion Criteria

The inclusion criteria stem directly from the review questions and were identified using the “Population, Concept, Context” framework recommended by JBI [28]. The population included adolescents ages 13 through 17. Adolescents were selected because there is an evident change in eating habits between adolescence and adulthood [29]. Thus, those aged 12 or younger and 18 or older fell outside of the scope of this review.

Snacking was defined as eating foods or consuming caloric beverages between meals, and SES was defined as household income and parental education level [30,31,32]. Weight-related outcomes in this review included weight gain, weight loss, BMI measures, overweight, obesity, underweight, weight change, weight status, lean mass, fat mass, percent body fat, or percent lean mass [33,34,35].

### 2.3. Search Strategies and Information Sources

A health sciences librarian (J.M.D.) developed the search strategy using the specified inclusion and exclusion criteria. Initial test searching was conducted in April 2019 through the databases Cumulative Index of Nursing and Allied Health Literature (CINAHL, EBSCO) and PubMed. Following expert team feedback and the refinement of keywords (see Appendix A (Table A1)) and subject headings (CINAHL Subject Headings and Medical Subject Headings), the base search strategy was peer-reviewed by another health sciences librarian and translated into 12 additional databases. The final searches occurred on 28–30 May 2019, using available subject headings, truncation, and phrase-searching in title and abstract fields. The following databases were selected due to their topic coverage of health, education, and the social sciences: EBSCO’s Academic Search Premier, CINAHL, Psychology and Behavioral Sciences Collection, PsycINFO, SPORTDiscus; Elsevier’s EMBASE; ProQuest’s Applied Social Sciences Index Abstracts (ASSIA), Education Resources Information Center (ERIC), International Bibliography of the Social Sciences (IBSS), Social Science Database, Sociological Abstracts, Sociology Database; PubMed, and Web of Science. Given the marked increases in obesity in recent decades, data was sought that was relevant to today’s populations and demographics. Thus, the results were limited to publication dates 2000 through May 2019 and to studies published in English, Portuguese, and Spanish languages, as these are the primary languages for the three title and abstract reviewers (V.G.W., A.D., J.R.D.). By including studies published in Portuguese and Spanish in addition to English, additional references were able to be screened. The results, however, were not limited to type of research design; all studies were included in the initial screening if they fit the inclusion criteria below. The search strategy for PubMed is located in Appendix A (Table A1), and the full search strategies are available from the librarian on request.

### 2.4. Study Screening, Study Selection, and Data Extraction

The literature searches retrieved 989 records in total, which were exported into Endnote Web and deduplicated. After deduplication, the 643 unique references were uploaded to Covidence for screening. Three reviewers (V.G.W., A.D., J.R.D.) independently screened the titles and abstracts from within Covidence (Table 1); screening disagreements were resolved through discussion with a third reviewer. 

If statistical analysis for the relationships between SES and snacking were included in the study, that information has been provided in this review [36,37,38,39]. For manuscripts that did not perform a statistical analysis on the relationship between SES and snacking but discussed that relationship in another manner [40,41,42], the data included by the authors in their publication as well as their interpretations of the observed data, if provided, have been reported in this review. It should be noted that although study design was not an exclusion criterion, the final 7 studies included for extraction were cross-sectional studies.

**Figure 1:**
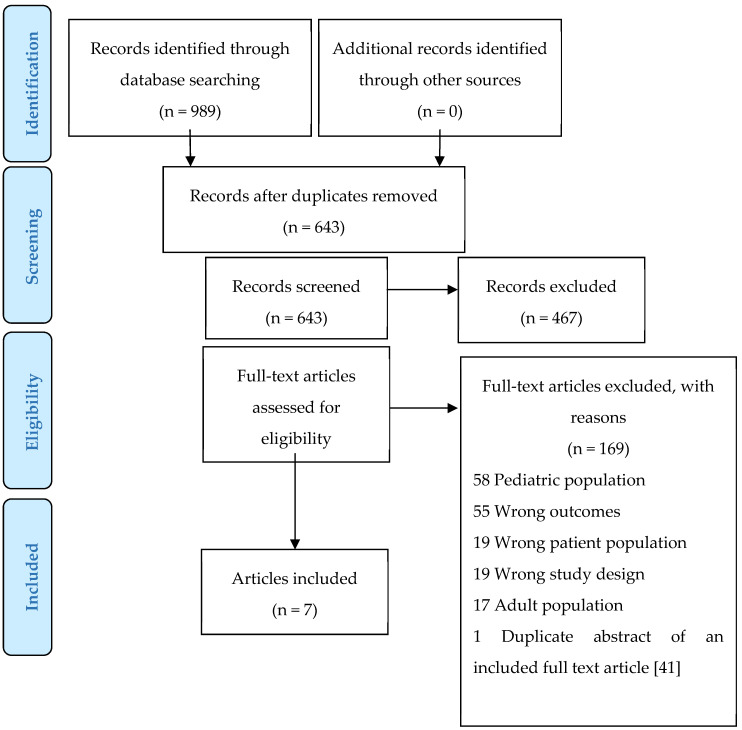
PRISMA (Preferred Reporting Items for Systematic Reviews and Meta-Analyses) Flow Diagram.

As detailed in Figure 1 in the Preferred Reporting Items for Systematic Reviews and Meta-Analyses (PRISMA) Flow Diagram, which was based on the 2009 PRISMA Statement for Reporting Items [43], 176 records progressed to full-text screening, with only 7 studies proceeding to the data extraction stage. Data extraction occurred using a standardized data charting form adapted from the JBI Reviewers Manual (Appendix B (Table A2)). Three reviewers read each full-text paper independently and conducted data extraction (Table 2 and Table 3).

## 3. Results

### 3.1. Low Socioeconomic Status and Higher Snacking

Two of the seven papers extracted found evidence of a relationship between low SES and high levels of snacking [40,42]. In a 2013 study by Grenard et al. conducted in the US, normal dietary patterns of high school students (n = 158) primarily from low SES homes were assessed by responding to questions involving food and beverage consumption at random, in the evening time, and right after eating or drinking [40]. In total, 49.0% of adolescents regularly consumed sweet or salty snacks; 16.5% regularly consumed salty snacks, and 36.1% regularly consumed sweet snacks. A 2014 study in Australia by Schumacher et al. sampled female adolescents (n = 332) from schools in communities with low SES and also found high snacking trends among their cohort [42]. On average, 6.8% of these adolescents’ daily calories were from calorie dense, nutrient-poor packaged snacks and these adolescents consumed ~1.5 snacks per day, on average. However, these percentages and daily snack consumption averages did not vary by BMI z-scores (i.e., weight status). Both of these studies that found this relationship, however, used a sample with low SES; there was not a control group and, thus, the conclusions drawn from these two studies are limited.

### 3.2. High Socioeconomic Status’ Variable Effect on Snack Consumption

We observed mixed results regarding the relationship of high SES with snack consumption among adolescents [37,38,39]. A 2015 study in the US by Larson et al. assessed meal and snack consumption among American adolescents (n = 2598 and n = 2540) in 1999 and 2010 [37]. These participants were a part of Project EAT in 1999 and EAT 2010 in 2010 which assessed dietary intake. This study concluded that SES does not have a statistically significant effect on snack intake.

A 2016 study in Ecuador conducted by Verstraeten et al. also found that SES had no effect on snacking [39]. Ecuadorian adolescents (n = 751) were classified into two SES categories: “poor” (i.e., lower SES) and “better off” (i.e., higher SES). These two categories were determined using the Integrated Social Indicator System in Ecuador, which is based on education, housing, employment opportunities, and other common SES factors. In total, 55% of adolescents in the sample were categorized into the “poor” group and 45% were categorized into the “high” group. SES was not significantly associated with unhealthy snacking in this Ecuadorian sample of adolescents. 

A 2011 study conducted in Africa by Maruapula et al. presents a different picture regarding the relationship between SES and snacking [38]. Snacking behavior among adolescents from public schools (indicating low SES in this sample; n = 492) and adolescents from private schools (indicating high SES in this sample; n = 212) in Botswana were assessed. In addition to using public or private school enrollment as an indicator of SES, number of possessions was also used to determine SES: 68.2% of the sample had a significant number of possessions, and 29.0% had a restricted number of possessions. Snack foods were determined to be foods with a high quantity of fat and sugar. Adolescents in the group with higher SES reported more daily servings of snacks than that of the group with lower SES (*p* < 0.01). Private school adolescents in the high SES category consumed more savory snacks (1.00 servings/day vs. 0.41 servings/day), sweet snacks (0.71 servings/day vs. 0.40 servings/day), and a higher quantity of snacks (2.14 servings/day vs. 0.92 servings/day) when compared to public school adolescents in the lower SES category. These snacking behaviors were also significantly correlated to increased prevalence of OW/OB. Thus, this study suggests that adolescent students from higher SES backgrounds consumed a higher quantity of snacks (both savory and sweet) and presented greater risks for developing obesity compared to students from lower SES backgrounds in Botswana.

### 3.3. Sex Differences in Socioeconomic Status and Snack Consumption

Only two of the seven studies investigated the relationship between snacking and sex, and both found that snacking behaviors differed by sex [36,41]. In a 2012 study using an Australian sample (n = 1568), Hardy et al. aimed to determine the prevalence and SES distributions of various obesogenic behavioral risk factors—one of which included high snack intake [36]. In this sample, adolescent males from lower SES backgrounds had significantly higher rates of snacking. On the contrary, the female adolescents from higher SES backgrounds had significantly higher rates of snacking. Thus, SES had differential impacts on snacking behavior by sex of Australian adolescents.

In the 2013 study in Spain by Pérez et al., dietary behaviors of 13–16 years old adolescents (n = 1620 for 3–16 aged sample) were collected via questionnaires [41]. Family socioeconomic level (FSEL) was determined by parental working status and professional category. In this study, higher SES categories were generally associated with less snacking (e.g., 28.3% of 13–16 years old males in FSEL quartile 4 consumed snacks daily vs. 49.4% of 13–16 years old males in FSEL quartile 1). Pérez and his colleagues also found that there is a difference in habits of snacking based on sex. In males aged 13–16, the habit of snacking decreases as FSEL increases, except in quartile 2—in which, snacking increases (Q1 49.4%, Q2 55.0%, Q3 43.1%, Q4 28.3%). In females aged 13–16, the habit of snacking decreases from 48.8% in the first FSEL quartile to 35.7% in the second FSEL quartile but increases to 38.5% in the third FSEL quartile and increases again to 47.2% in the fourth FSEL quartile. Thus, this paper suggests that SES may differentially impact snacking behavior among Spanish adolescent males and females.

### 3.4. Weight-Related Outcomes Related to Snacking and SES

Only three of the seven studies analyzed weight-related outcomes and the observed relationship between snacking and weight differed between the studies [36,38,42]. In the study by Maruapula et al., adolescent students in higher SES categories had higher snacking rates and higher rates of OW/OB [38]. In a high snacking diet, rates of OW/OB significantly increased; however, this relationship was no longer significant once the authors controlled for SES in the analysis. In the Hardy et al. study, a smaller proportion of male adolescents with OW/OB reported high snacking when compared to male adolescents without OW/OB (*p* < 0.001); however, Hardy and colleagues did not find a significant relationship between snacking and weight among female adolescents [36]. This aligns with results from another study by Schumacher et al., which showed no correlation between snacking and risks for OW/OB in female adolescents with low SES [42]. Thus, low SES and high rates of snacking did not have an association with OW/OB.

## 4. Discussion

### 4.1. Primary Aims: Relationship between SES and Snacking

This review characterized the relationship between SES, snacking, and weight-related outcomes. Based on the findings, we also explored possible differences with sex and income type of country. With studies from every habitable continent, this research is relevant on a global scale. However, the evidence for a potential association between SES and snacking behavior is weak given the mixed results among the included studies and the paucity of between group analyses comparing snacking behavior in groups with low and high SES. The results provide modest evidence suggesting that snacking behaviors may differ based on sex. Additionally, the currently available data does not support the notion that snacking plays a significant role in weight-related outcomes after controlling for SES. Interesting to note, the relationship between SES and snacking appears to differ based on region and income status of the country, as results were inconsistent between the high-income countries and middle-income countries included in this review. However, all results should be interpreted with a great deal of caution given significant variability between studies and the small number of studies included in the review. A causal relationship between SES, snacking, and weight-related outcomes cannot be concluded as the final included studies were cross-sectional and observational. The main conclusion drawn from this review is that additional research is needed to characterize the relationships among SES, snacking, and weight-related outcomes among adolescents as the currently available literature is sparse.

Two of the studies supported a relationship between low SES and high snacking [40,42], with adolescents with low SES reporting regular consumption of sweet or salty snacks, and almost 7% of daily calories derived from calorie dense, nutrient-poor packaged snacks [40,42]. However, this was only present in two studies, both of which only included a sample with low SES and thus no control group for comparison, limiting the inferences that can be made from these results. Therefore, caution should be exercised in generalizing these results. The relationship between high SES and snack outcomes was much less clear. Snacking behaviors related to high SES were variable, as one study found that high SES was associated with increased snack consumption [38] and two others found that high SES was not significantly correlated with snack consumption [37,39]. Taken together, these results provide a weak potential link between SES and snacking behaviors. These results are consistent with the varying results found in the literature on the impacts of SES on eating behaviors around the world [44,45,46]. However, upon further investigation of the results and separating them between income economy type (middle and high), different trends emerge that provide some insight for these findings. 

Among studies from high-income countries, two studies found that low SES was associated with increased snack consumption [40,42], and one found no significant association between high SES and snack consumption [37]. When separating these results based on income economy type, there is a possible trend between lower SES and increased snacking in high-income economies. This aligns with a large body of literature from high-income economies—in which, low SES has been linked with poorer nutritional patterns when compared to groups with high SES [47,48,49]. It is important to note that these two studies identifying high levels of snacking in groups with low SES did not compare groups with low SES to groups with high SES; these studies merely found high levels of snacking in their low SES sample without a control or comparison [40,42]. Thus, the inferences that can be drawn from these studies are limited. In high-income economies like the US, Spain, and Australia, this relationship can be explained by a lack of access to healthy foods due to their high price [50,51]; greater availability of fast food restaurants where energy dense, nutrient-poor foods are accessible and affordable [52,53,54]; lack of access to healthy foods as low-income households are less likely to have vehicles for grocery shopping [55]; and the lack of large grocery stores where fresh produce is available in low SES neighborhoods [54,55,56]. Finally, there is evidence that individuals in lower SES environments have greater perceived stress [57] and that individuals under psychological stress consume higher quantities of energy-dense snacks [58]. Additionally, studies that have experimentally manipulated social status in a laboratory setting find that low social status is associated with increased calorie consumption [24,25]. However, not all studies from high-income countries included in this review found a significant association between snacking and SES; thus, the support for this relationship remains very weak.

Among the two studies from middle-income economies, results on the relationship between SES and snacking were variable, with one study finding that SES had no significant effect on snacking and one finding that higher SES was associated with higher quantities of snacking. Thus, these results suggest that more research is needed in in middle-income economies to quantify the trends between SES and snacking. Despite the variability in these results, they provide evidence that snacking behaviors may differ between middle- and high-income economies with middle-income economies aligning more closely to the relationship between high SES and high snacking than the low SES and high snacking relationship which may be more representative of high-income economies. Of particular interest, in their study based in Ecuador, Verstraeten and co-authors describe behavior markedly different from results found in the US. In this study, a better understanding of both the importance of healthy eating and understanding of healthy food were associated with a higher intake of unhealthy snacks [39]. Additionally, higher perceived benefits of healthy eating were positively and directly associated with increased unhealthy snacking [39]. This differs from the established relationship between health literacy and positive diet quality in high-income economies like the US and Japan [59,60]. The second study from a middle-income economy was conducted in Botswana, Africa, and was the only study which found that higher SES was significantly associated with high quantity of snacks and/or increased unhealthy snacking. Groups with high SES in this region may have greater access to Western snack foods, which are typically energy-dense, and this could be contributing to the association between groups with high SES and OW/OB [38]. This notion is supported by childhood OW/OB prevalence data in middle- and low-income countries that show a reverse relationship from OW/OB trends in high-income countries [61,62]. All of the studies that support the relationship between low SES and high snacking come from high-income economies. Since Botswana, like Ecuador, is an upper middle-income economy, it could be that such interactions with snacking function differently in different economies. As Maruapula and co-authors point out, the different patterns in snack behaviors by SES and the high rates of OW/OB noticed in Botswana can be at least partially explained by the country undergoing a period of nutritional transition [38]. Maruapula et al. argue that Botswana is currently in the receding famine phase of the nutritional transition, having not yet reached the phase of behavioral change [38]. This limits the generalizability of this finding. 

Two of the studies, both from high-income economies, found results that differed based on sex [36,41]. These results align with research indicating that males and females, both adults and children, have different snacking preferences and behaviors. For example, male adolescents have been shown to consume more energy dense fruits and vegetables [63] and snacks and sugar sweetened beverages [64]. Male adolescents have also been shown to prefer meat, poultry, and fish over female adolescents, and female adolescents have been shown to prefer fruits and vegetables over male adolescents [65]. However, more research is needed specifically on sex differences within the relationship between SES and dietary intake to understand the sex-based differences displayed within these two studies. 

The overall results of this paper somewhat align with similar studies conducted among adult populations. In a study conducted in the US using data of individuals aged 19–65 from the National Health and Nutrition Examination Surveys, the income-to-needs ratio was positively associated with the accessibility of salty snacks in the home and college graduates were less likely to consume salty snacks than their counterparts who did not graduate from college [66]. Another study conducted in Suriname found a higher prevalence of excessive intakes of snacks, fast food meals, and sweets among those living in urban areas and with higher SES [67]. Like Maruapula et al., Nahar-van Venrooij et al. argue that this trend of high snacking among high SES individuals in Suriname may be due to the middle-income country’s position in the nutrition transition, with individuals with high SES in low-income and middle-income countries being affected by the nutrition transition before those with lower SES [67]. One recent study conducted in France found that snack consumption varied by SES indicator [68]. For example, those with lower education levels were less likely to snack whereas there was no significant difference in weekday snacking by income level [68]. Taken together, these results indicate that the impact of SES on snacking behaviors may be somewhat similar between adolescent and adult populations. However, due to the paucity of research on adolescent populations, more research is needed to characterize the extent to which adolescent and adult populations are similar or different in their snacking behaviors.

### 4.2. Secondary Aims: Weight-Related Outcomes

The relationship between snacking and weight-related outcomes in the literature has been relatively inconsistent [69], with some studies finding positive relationships [10,70], inverse relationships [71,72], or no relationships [73,74,75,76]. Our secondary aims, which were to assess the relationship between snacking and weight-related outcomes among adolescents, were only addressed by three of the included studies. These studies, from Botswana and Australia, provide evidence that snacking level was not significantly associated with weight-related outcomes after controlling for SES. These results suggesting that snacking does not significantly affect weight-related outcomes across middle-and high-income economies, aligning with many studies of diverse groups of males and females that have also found no significant association between snacking weight-related outcomes [77,78,79]. Potential explanations for this non-significant association could be that most of the included studies focused on quantity of snack consumption [36,42] and not quality of snack consumption. A recent review emphasizes the importance of snack quality over snack quantity in determining weight-related outcomes [69]. Healthy snacking, Hess et al. argues, likely impacts satiety levels and stimulates appetite control, which could decrease risk for OW/OB [69]. Importantly, in Hardy et al.’s study, male adolescents did not present the same weight-related results as female adolescents [36]. These results provide preliminary evidence that the effects of snacking on OW/OB could differ based on sex. More research should be conducted to understand this relationship. Of particular interest, Hardy and co-authors found that adolescent males without OW/OB reported higher snacking than adolescent males with OW/OB, suggesting that snacking does not contribute as much to OW/OB as other eating behaviors, such as low fruit and vegetable intake and high soft drink intake, both of which were significantly associated with OW/OB among females in this study [36]. This aligns with other research suggesting that energy-rich, nutrient-poor foods and sugar sweetened soft drinks are among the most significant contributors of OW/OB [80]. Overall, weight-related outcomes were only addressed by three of the included studies [36,38,42] and while results from these three studies do not necessarily indicate a significant relationship with weight-related outcomes, due to the limited nature of this review and the small number of studies addressing this question, we urge readers to keep these limitations in mind when interpreting these results.

Overall, the results from the studies presented in this review suggest that it is unlikely that snacking significantly impacts OW/OB among adolescents in middle-and high-income economies. However, due to the very low number of included studies investigating this relationship and the slight variance in the presented results, we advise exercising caution when interpreting the results of weight-related outcomes in these included studies. We recommend that this under researched topic be included in future studies to investigate the impact of SES on snacking behaviors among adolescents.

### 4.3. Limitations

One weakness of this review is the potential exclusion of many studies based on our rigorous inclusion and exclusion criteria. For example, many studies including participants inside and outside of our age range that also did not report a mean/median age were excluded, since no calculation was possible. Similarly, for the purpose of this study, papers had to refer to snack consumption explicitly as “snacks” or defined as “foods or beverages consumed between meals”. Some papers were excluded because snack consumption was embedded in a larger dietary pattern like a “modern diet” or paired snacks with sugar sweetened beverages and/or desserts—in which, the authors did not define the latter foods as being consumed between meals.

Additionally, results regarding the region-specific associations may be biased due to the language of the studies that were included. While researchers attempted to be as culturally sensitive as possible, by allowing studies written in English, Portuguese, and Spanish to be reviewed and/or included, it was not possible to review or include studies written in other languages (e.g., Russian or French), due to limitations in the coauthors’ language proficiency.

It should also be noted that results from this review cannot determine causality within the interactions between SES, snacking, and weight-related outcomes due to the observational nature of all the studies selected for inclusion. Additionally, two of the seven included studies were conducted in a population with low SES, without a control group, limiting the relationships that can be derived from that data. Future research employing longitudinal designs and experimentally manipulated paradigms may address this question and offer novel insights into mechanisms underlying these relationships. Additionally, as this is a scoping review, no ratings of the quality of the sources, strength of evidence, or rating of bias assessment within each source is provided. Factors such as strength of evidence should be taken into consideration when developing best practices or implementing relevant policies.

## 5. Conclusions and Recommendations

### 5.1. Conclusions

In conclusion, this review suggests that the relationship between SES and snacking among adolescents may function differently in different cultures, geographical regions, and income economies. For example, among high-income economies, this review suggests there may be a trend for low SES to be associated with increased snack consumption and adverse snacking behaviors. On the contrary, middle-income economies seem to lean more towards an association between high SES and increased snack consumption. It should be noted that region-specific conclusions drawn from included studies may be biased due to the limitation of reviewing studies written in English, Portuguese, or Spanish, only. However, there is not enough evidence presented in the included studies to determine the strength of the relationship, if any, between SES and snacking. While the relevant weight-related outcomes resulting from these relationships are less clear than the primary outcomes, most of the results appear to support that snacking does not have a significant effect on weight-related outcomes. However, due to the very small number of studies included in this review, results from this review should be interpreted with caution.

### 5.2. Recommendations for Research

No studies from low-income economies were included in this review. This may be because studies of this scope were excluded in our rigorous inclusion/exclusion criteria, or it could be that fewer studies of this scope are conducted in low-income economies. If the latter is true and given our results suggesting these relationships possibly differ based on economy status, it is particularly important that studies assessing relationships between SES, snacking, and weight among adolescents be conducted in low-income economies, as they may reveal novel relationships that differ from those in middle-income and high-income economies. For example, Maruapula et al.’s 2011 study, which found results that contradicted the most supported results in our primary aims, was the first of its kind to examine the role of SES on dietary intake patterns among adolescents in Botswana [38]. Similarly, more research should also be conducted on middle-income economies, as the two studies included in this review did not align with our primary aim outcomes and, thus, may require different public health intervention efforts from those utilized in high-income countries. Furthermore, only three studies looked at weight-related outcomes related to snacking, with minimal evidence that SES and snacking relates to weight. More studies on the impact of snacking and SES on weight-related outcomes among adolescents should be conducted to further answer this question. Finally, future research should employ experimental paradigms and longitudinal designs to further explain these patterns, including the causal mechanisms underlying these relationships.

### 5.3. Recommendations for Practice

Due to the variation in agreement among our findings, there is not enough evidence to make recommendations for clinical practice at this time. However, preliminary evidence from this review suggests that when attempting to change dietary patterns among adolescents, healthcare practitioners may benefit by considering SES as a factor that influences patients’ dietary habits, including snacking. 

## Figures and Tables

**Table 1:** Screening Inclusion and Exclusion Criteria.

Inclusion Criteria	Exclusion Criteria
1. Must include information regarding socioeconomic status (SES) (including education and/or income) and snacking; weight-related outcomes are secondary	1. Language other than English, Portuguese, or Spanish
2. Snacking behavior was assessed	2. Publication year before 2000
3. Mean or median age of participants must fall within 13–17. If the mean and median are both presented, the median is used as the determining vote	3. Mean or median age of participants falls outside of 13–17
	4. The primary population (defined as more than half of the study participants) includes adolescents with a significant medical diagnosis, such as disordered eating, substance use or abuse, cancer, cardiovascular disease, diabetes, ADHD, autism, or any other disease state for which they are administered medication that is known to affect eating behavior, weight, or body composition
	5. Opinion paper, editorial, or policy and legislation literature
	6. Does not assess or discuss the relationship between SES and snacking
	7. If the variable for snacking is not snack consumption (e.g., snack preference, snack choice, but no evidence of consumption)

**Table 2:** Description of Included Studies.

Study Authors (Year); Country	Sample Size (n)	Age Range (years); Mean or Median (Years)	Study Design; Duration	Setting
Grenard et al. (2013) [40]; US	158	Range = 14–17; mean age = 15.98	Cross-sectional study; 7 days	Various participant locations in the US
Hardy et al. (2012) [36]; Australia	1568	No age range reported; 8th grade mean age = 13.6; 10th grade mean age = 15.6; overall mean age = 13.74	Cross-sectional study; 4 months	Primary and secondary schools in New South Wales, Australia
Larson et al. (2015) [37]; US	2598 (1999); 2540 (2010)	No age range reported; mean age = 14.62 (1999) and 14.52 (2010)	Repeated, cross-sectional study; two separate academic years (1999 and 2010)	Selected health, physical education, and science classes in secondary schools located in Minneapolis-St. Paul, MN, US
Maruapula et al. (2011) [38]; Botswana	704	No age range reported; mean age = 14.9	Nationwide, cross-sectional study	Secondary schools in Botswana, Africa
Pérez et al. (2013) [41]; Spain	1620	Range = 3–16 (subsample range = 13–16); no mean age reported	Cross-sectional study; one-time visit	Schools in Cádiz, Spain
Schumacher et al. (2014) [42]; Australia	332	Range = 13.4–13.9; mean age = 13.7	Secondary analysis of Nutrition and Enjoyable Activity for Teen Girls (NEAT Girls) Randomized Controlled Trial (RCT); 12 months	Governmental, low-income secondary schools in the Hunter and Central Coast regions of New South Wales, Australia
Verstraeten et al. (2016) [39]; Ecuador	751	Range = 10–16; mean age = 13.6	Cross-sectional study; 16 months	Cuenca and Nabón, Ecuador

**Table 3:** Study Measures and Outcomes.

Study Authors	SES of Cohort	Observed Snacking Behaviors	Weight Outcomes	Assessments
Grenard et al. [40]	Low SES	High consumption of calorie-dense snacks in low SES sample (16.5% salty; 36.1% sweet).	Not assessed.	SES was self-reported via a questionnaire; snacking was measured via one-to-one interviews at baseline and through self-initiated and random eating event reports during the 7 day monitoring period.
Hardy et al. [36]	Various SES groups	Male adolescents with lower SES have high rates of snacking (*p* = 0.045); female adolescents with higher SES have high rates of snacking (*p* = 0.034).	Smaller portion of male adolescents with overweight (OW)/obesity (OB) reported higher snacking than male adolescents without OW/OB (*p* < 0.001); no significant weight-related outcomes associated with snacking in female adolescents (*p* = 0.235).	Parents completed questionnaire with mother’s educational attainment and household income; dietary information was collected using questions based on the NSW Population Health Surveys’ food frequency questionnaire (FFQ). BMI was calculated for each participant using height and weight and OW/OB categorization was conducted via the International Obesity Task Force standards.
Larson et al. [37]	Various SES groups	High–medium and high SES categories had low consumption of snacks.	Not assessed.	SES was self-reported via survey; Youth and Adolescent FFQ was used to assess adolescent snack consumption.
Maruapula et al. [38]	Low and high SES groups	Higher SES adolescents consumed a larger quantity of snacks (*p* < 0.01).	High snack food diet was found to increase risk for OW/OB (*p* = 0.028); however, SES was not a significant determinant of OW/OB.	SES determined by attendance at tuition-free public or tuition-requiring private school and by the number of household assets. A single portable precision electronic scale and stadiometer were used to obtain anthropometric measurements; participants self-recorded their recall of food intake for the previous day. BMI was calculated for each participant using height and weight and was evaluated using the World Health Organization’s reference data for age and gender.
Pérez et al. [41]	Low, middle, and high SES groups	In males, the habit of snacking decreases as SES increases, except in quartile 2—in which, snacking increases. In females, the habit of snacking increases, except from the first to second quartile—in which, snacking decreases. Quartile 1 has the highest snacking habits of any of the other SES quartiles.	Not assessed.	SES determined using the family socioeconomic level (FSEL); snack consumption measured using 24 h dietary recall and food frequency questionnaire adaptation of the short questionnaire on frequency of dietary intake.
Schumacher et al. [42]	Low SES	Evidence of high snack consumption. In total, 6.8% of daily energy from snacks. Average of 1.5 snacks per day.	Not a significant association (*p* = 0.47).	The Australian Child and Adolescent Eating Survey (ACAES, version 1.2), an FFQ estimated dietary intake data over the previous 6 months; measured height and weight. BMI was calculated for each participant and then ranked into underweight, healthy, overweight, or obese categories.
Verstraeten et al. [39]	“Poor” SES and “better off” SES groups	No significant association between low SES and unhealthy snack consumption.	Not assessed.	SES was measured using the Integrated Social Indicator System for Ecuador. Then, participants classified as “poor” or “better off”; snack consumption measured using 24 h dietary recall interviews.

## Appendix A

**Table A1:** Sample Search Strategy.

PubMed Search Strategy, Searched on 28 May 2019	
Set Number	Search Terms
1	(“Socioeconomic Factors”[Mesh] OR socioeconomic*[tiab] OR “SES”[tiab] OR “social economic”[tiab] OR “social economics”[tiab] OR “social economical”[tiab] OR “social economically”[tiab] OR “social stratification”[tiab] OR “social class”[tiab] OR “social classes”[tiab] OR “social stratum”[tiab] OR “social stratifications”[tiab] OR “social strata”[tiab] OR “social status”[tiab] OR “social statuses”[tiab] OR “SSS”[tiab] OR “social factors”[tiab] OR “economic factors”[tiab] OR “middle class”[tiab] OR “middle classes”[tiab] OR “lower class”[tiab] OR “lower classes”[tiab] OR “working class”[tiab] OR “working classes”[tiab] OR “upper class”[tiab] OR “upper classes”[tiab] OR “social caste”[tiab] OR “social castes”[tiab] OR “standard of living”[tiab] OR “living standard”[tiab] OR “living standards”[tiab] OR “upper income”[tiab] OR “upper incomes”[tiab] OR “high income”[tiab] OR “high incomes”[tiab] OR “low income”[tiab] OR “low incomes”[tiab] OR “income level”[tiab] OR “income levels”[tiab] OR “higher income”[tiab] OR “higher incomes”[tiab] OR “lower income”[tiab] OR “lower incomes”[tiab] OR “social inequality”[tiab] OR “social inequalities”[tiab] OR “social equity”[tiab] OR “economic class”[tiab] OR “economic classes”[tiab] OR “economic stratum”[tiab] OR “economic stratification”[tiab] OR “economic strata”[tiab] OR “economic status”[tiab] OR “economic statuses”[tiab] OR “economic inequality”[tiab] OR “economic inequalities”[tiab] OR “social condition”[tiab] OR “social conditions”[tiab] OR “living condition”[tiab] OR “living conditions”[tiab] OR “social mobility”[tiab] OR “social mobilities”[tiab] OR “household income”[tiab] OR “household incomes”[tiab] OR “household wealth”[tiab] OR “inherited wealth”[tiab] OR “home ownership”[tiab] OR “property ownership”[tiab] OR poverty[tiab] OR indigen*[tiab] OR homeless*[tiab] OR “economic deprivation”[tiab] OR “economically deprived”[tiab] OR “social deprivation”[tiab] OR “financial strain”[tiab] OR “financial strains”[tiab] OR “debt level”[tiab] OR “debt levels”[tiab] OR “level of debt”[tiab] OR “income gap”[tiab] OR “income gaps”[tiab] OR unemployed[tiab] OR underemployed[tiab] OR “economically disadvantaged”[tiab] OR “socially disadvantaged“[tiab] OR “economically secure”[tiab] OR “economic security”[tiab] OR “material wealth”[tiab] OR underprivileg*[tiab])
2	((literate[tiab] OR literacy[tiab] OR illitera*[tiab] OR “education level”[tiab] OR “educational level”[tiab] OR “education levels”[tiab] OR “educational levels”[tiab] OR “educational status”[tiab] OR “education status”[tiab] OR “level of education”[tiab] OR “educational attainment”[tiab] OR “education background”[tiab] OR “education backgrounds”[tiab] OR “educational background”[tiab] OR “educational backgrounds”[tiab] OR graduate*[tiab] OR “educational achievement”[tiab] OR “educational achievements”[tiab] OR “academic achievement”[tiab] OR “academic achievements”[tiab] OR “academic attainment”[tiab] OR “educationally disadvantaged”[tiab] OR “educational stratification”[tiab] OR “achievement gap”[tiab] OR “achievement gaps”[tiab] OR schooling[tiab] OR “school years complete”[tiab] OR “school years completed”[tiab]) AND (parent*[tiab] OR family[tiab] OR families[tiab] OR mother[tiab] OR mothers[tiab] OR father[tiab] OR fathers[tiab] OR mum[tiab] OR mums[tiab] OR dad[tiab] OR dads[tiab] OR mom[tiab] OR moms[tiab] OR paternal[tiab] OR maternal[tiab] OR birthparent*[tiab] OR birthmother*[tiab] OR birthfather*[tiab]))
3	#1 OR #2
4	(“Adolescent”[Mesh] OR “Adolescent Nutritional Physiological Phenomena”[Mesh] OR “Adolescent Development”[Mesh] OR “Adolescent Health”[Mesh] OR “Adolescent Health Services”[Mesh] OR “Adolescent Behavior”[Mesh:NoExp] OR “Adolescent Medicine”[Mesh] OR “Adolescent Psychiatry”[Mesh] OR “Psychology, Adolescent”[Mesh] OR adolescen*[tiab] OR teen*[tiab] OR youth[tiab] OR youths[tiab] OR youngster*[tiab] OR juvenile*[tiab] OR pubescent[tiab])
5	(“Snacks”[Mesh] OR snack*[tiab] OR “intake between meals”[tiab] OR “consumption between meals”[tiab] OR “eating between meals”[tiab] OR “eat between meals”[tiab] OR “eats between meals”[tiab] OR “food between meals”[tiab])
6	(“Body Weight”[Mesh:NoExp] OR “Ideal Body Weight”[Mesh] OR “Body Weight Changes”[Mesh] OR “Overweight”[Mesh] OR “Thinness”[Mesh] OR “Body Weight Maintenance”[Mesh] OR “Body Mass Index”[Mesh] OR “Body Fat Distribution”[Mesh] OR weight[tiab] OR weights[tiab] OR “body mass index”[tiab] OR “BMI”[tiab] OR “BMIs”[tiab] OR “BMIZ”[tiab] OR “body fat”[tiab] OR “body fatness”[tiab] OR obese[tiab] OR obesity[tiab] OR overweight[tiab] OR thinness[tiab] OR emaciation[tiab] OR emaciated[tiab] OR “excess fat”[tiab] OR “excess fats”[tiab] OR adiposity[tiab] OR “BAI”[tiab] OR underweight[tiab] OR “quetelet index”[tiab] OR “lean mass”[tiab] OR “fat mass”[tiab])
7	#3 AND #4 AND #5 AND #6
8	#7, Filters: English, Portuguese, Spanish; Publication date from 2000/01/01

## Appendix B

**Table A2:** Data Charting Form.

a. Author(s)
b. Year of publication
c. Origin/country of origin (where the study was published or conducted)
d. Aims/purpose
e. Study population and sample size (if applicable)
f. Methods (qualitative, quantitative, or mixed)
g. Results for primary aim
h. Results for secondary aim
i. Implications of results for the purpose of this scoping review
